# Pathway engineering for high-yield production of lutein in Escherichia coli

**DOI:** 10.1093/synbio/ysab012

**Published:** 2021-05-15

**Authors:** Miho Takemura, Akiko Kubo, Asuka Watanabe, Hanayo Sakuno, Yuka Minobe, Takehiko Sahara, Masahiro Murata, Michihiro Araki, Hisashi Harada, Yoshinobu Terada, Katsuro Yaoi, Kohji Ohdan, Norihiko Misawa

**Affiliations:** Research Institute for Bioresources and Biotechnology, Ishikawa Prefectural University, Nonoichi, Ishikawa, Japan; Applied Research Laboratory, Ezaki Glico Co., Ltd., Osaka, Japan; Research Institute for Bioresources and Biotechnology, Ishikawa Prefectural University, Nonoichi, Ishikawa, Japan; Research Institute for Bioresources and Biotechnology, Ishikawa Prefectural University, Nonoichi, Ishikawa, Japan; Research Institute for Bioresources and Biotechnology, Ishikawa Prefectural University, Nonoichi, Ishikawa, Japan; Bioproduction Research Institute, National Institute of Advanced Industrial Science and Technology (AIST), Tsukuba, Ibaraki, Japan; Graduate School of Medicine, Kyoto University, Kyoto, Japan; Graduate School of Medicine, Kyoto University, Kyoto, Japan; Faculty of Engineering, Tottori University, Tottori, Japan; Mechanism-Based Research Laboratory, Ezaki Glico Co., Ltd., Osaka, Japan; Bioproduction Research Institute, National Institute of Advanced Industrial Science and Technology (AIST), Tsukuba, Ibaraki, Japan; Applied Research Laboratory, Ezaki Glico Co., Ltd., Osaka, Japan; Research Institute for Bioresources and Biotechnology, Ishikawa Prefectural University, Nonoichi, Ishikawa, Japan

**Keywords:** lutein, *Escherichia coli*, pathway engineering, genome insertion, heterologous expression

## Abstract

Lutein is an industrially important carotenoid pigment, which is essential for photoprotection and photosynthesis in plants. Lutein is crucial for maintaining human health due to its protective ability from ocular diseases. However, its pathway engineering research has scarcely been performed for microbial production using heterologous hosts, such as *Escherichia coli*, since the engineering of multiple genes is required. These genes, which include tricky key carotenoid biosynthesis genes typically derived from plants, encode two sorts of cyclases (lycopene ε- and β-cyclase) and cytochrome P450 CYP97C. In this study, upstream genes effective for the increase in carotenoid amounts, such as isopentenyl diphosphate isomerase (*IDI*) gene, were integrated into the *E. coli* JM101 (DE3) genome. The most efficient set of the key genes (*MpLCYe, MpLCYb* and *MpCYP97C*) was selected from among the corresponding genes derived from various plant (or bacterial) species using *E. coli* that had accumulated carotenoid substrates. Furthermore, to optimize the production of lutein in *E. coli*, we introduced several sorts of plasmids that contained some of the multiple genes into the genome-inserted strain and compared lutein productivity. Finally, we achieved 11 mg/l as lutein yield using a mini jar. Here, the high-yield production of lutein was successfully performed using *E. coli* through approaches of pathway engineering. The findings obtained here should be a base reference for substantial lutein production with microorganisms in the future.

## 1. Introduction

Carotenoids are isoprenoids with a large number of conjugated double bonds that exhibit yellow to red coloration. The pigments are biosynthesized by all photosynthetic organisms composed of photosynthetic bacteria, cyanobacteria, algae and plants (1–3). Photosynthetic organisms must utilize carotenoids to fulfill light harvest in photosynthesis and to protect themselves from oxidative damage generated by excessive light (4, 5). The photosynthetic organs of land plant chloroplasts that include higher plants and liverworts retain lutein [(3*R*,3′*R*,6′*R*)-β, ε-carotene-3,3′-diol] as the predominant carotenoid (ordinarily ∼45% and ∼58% of the total carotenoids in higher plants and liverwort, respectively (6, 7), which is a crucial component of light-harvesting complex II) (8).

In humans, lutein exists in the macula lutea along with zeaxanthin owing to their dietary intake. Lutein is considered to protect the human eyes from photooxidative damage by filtering high-energy blue light and preventing ocular diseases such as age-related macular degeneration and cataract (9, 10). This pigment is also present in other human tissues and is suggested to protect the skin from ultraviolet-induced damage and to reduce the risk of cardiovascular diseases such as atherosclerosis (9, 11). Thus, lutein is now commercially produced for functional supplements and foods, typically by cultivating marigold and extracting the pigment from flowers. According to a BCC research report (12), lutein’s global market value is about 300 million dollars, which corresponds to the maximal amount among carotenoid species for sale.

Pathway engineering, which is a metabolic engineering with heterologous microbial hosts (13), has been arduously performed to efficiently produce several commercialized carotenoids, i.e. lycopene, β-carotene, zeaxanthin and astaxanthin, using *Escherichia coli,* representatively (e.g. 14–18). In such a pathway engineering research of these carotenoids, bacterial carotenoid biosynthesis (*crt*) genes have been utilized for their functional expression, e.g. *crtE, crtB, crtI, crtY, crtZ* and *crtW* genes have been used for astaxanthin production (15, 19, 20). Alternatively, few reports have described lutein biosynthesis in the *E. coli* host by the functional expression of carotenogenic genes that were derived from a plant (liverwort) (genes of lutein biosynthesis from lycopene) and bacteria (the *crtE, crtB* and *crtI* genes for lycopene biosynthesis) (21, 22).

**Figure 1.**
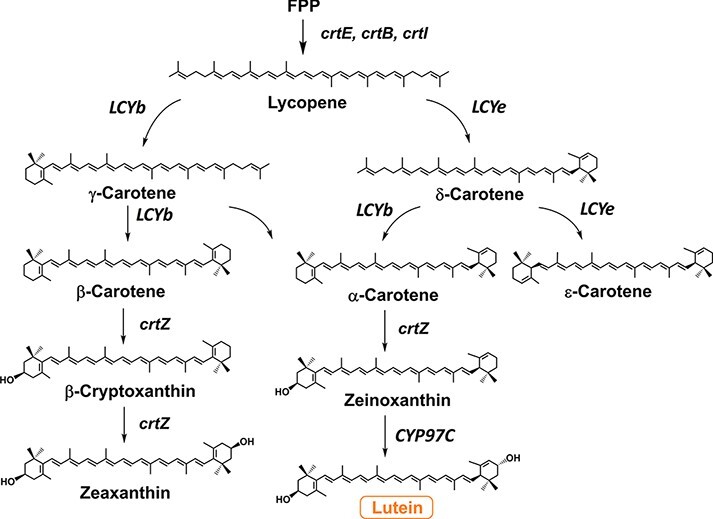
Lutein biosynthesis pathways in the recombinant *E. coli.* FPP, farnesyl pyrophosphate; *crtE:* GGPP synthase; *crtB*, phytoene synthase; *crtI*, carotenoid desaturase; *LCYb*, lycopene β-cyclase; *LCYe*, lycopene ε-cyclase; *crtZ*, β-carotene hydroxylase; *CYP97C*, cytochrome P450 97C.

This study shows the construction of a recombinant *E. coli* that produced lutein efficiently by engineering multiple genes derived from bacteria and a plant (liverwort) (Figure 1).

## 2. Materials and methods

### 2.1 Plasmid construction

Plasmids and *E. coli* strains used in this study are summarized in Supplementary Figure S1 and Supplementary Tables and S2. The sequences of the plasmids constructed in this study are provided in the Supplementary information. We constructed the plasmid pAC-HIEBI that contained the *Haematococcus pluvialis IDI* (IPP isomerase), *Pantoea ananatis crtE* (GGPP syntthase), *crtB* (phytoene synthase) and *crtI* (phytoene desaturase) genes (20; accession no. ERWCRT), flanked by the *tac* promoter (P*tac*) and the *rrnB* terminator (T*rrnB*), as described below. P*tac* and T*rrnB* fragments were amplified by the polymerase chain reaction (PCR) and inserted into the *Nco*I and *Eco*RII sites of an *E. coli* vector pACYC184. Next, *IDI, crtE, crtB* and *crtI* gene fragments were amplified by PCR, sequentially ligated and inserted into the *Xho*I and *Kpn*I sites between P*tac* and T*rrnB* of the above-constructed plasmid to construct the plasmid pAC-HIEBI. Similar to pAC-HIEBI, we constructed the plasmid pRK-HIEBI. P*tac* and T*rrnB* fragments were amplified by PCR and inserted into the *Hin*dIII and *Bgl*II sites of the *E. coli* vector pRK404, after which *IDI, crtE, crtB* and *crtI* gene fragments were amplified by PCR and inserted into the *Xho*I and *Kpn*I sites between P*tac* and T*rrnB* of the above-constructed plasmid to construct the plasmid pRK-HIEBI.

The coding region of the *LCYe* gene from *Marchantia polymorpha* (*MpLCYe*) was artificially synthesized with the codons optimized to those of *E. coli* K12 strain and then inserted into the plasmid pAC-HIEBI. This plasmid was named as pAC-HIEBI-MpLCYe. The accession number of the synthetic *MpLCYe* gene is LC603184.

The *crtYm* gene was amplified by PCR with the plasmid pBS603 as a template (23; accession no. AB097813). The *cruA* gene (accession no. NC_008639) of *Chlorobium phaeobacteroides* was artificially synthesized with modified codons optimized to *E. coli* K12. These fragments were then inserted into the pAC-HIEBI or pAC-HIEBI-MpLCYe, yielding the plasmids pAC-HIEBIYm, pAC-HIEBIA, pAC-HIEBIA-MpLCYe and pAC-HIEBI-MpLCYe-A.

The coding region of the *MpLCYb* gene was amplified with the plasmid pETD-MpLCYb/MpLCYe (7; accession no. AB794089) as a template and cloned into the pAC-HIEBI and pRK-HIEBI. The resulting plasmids were named as pAC-HIEBI-MpLCYb and pRK-HIEBI-MpLCYb. The transit peptide of the *MpLCYb* gene product was predicted by ChloroP (http://www.cbs.dtu.dk/services/ChloroP/). The truncated form of *MpLCYb* gene (*MpLCYbΔTP*) was then amplified by PCR and cloned into plasmids similar to MpLCYb. The codon-optimized MpLCYb (MpLCYbop) (accession no. LC603183) was chemically synthesized and used to construct the plasmid pAC-HIEBI-MpLCYbop.

The *P. ananatis crtZ* genes were amplified by PCR as well, ligated with the *MpLCYe* fragment and inserted into the plasmids pAC-HIEBI-MpLCYb(ΔTP, op) and pRK-HIEBI-MpLCYbΔTP to construct pAC-HIEBI-MpLCYb(ΔTP, op)-MpLCYe-Z and pRK-HIEBI-MpLCYbΔTP-MpLCYe-Z, respectively.

The coding regions of the *LCYe* genes from *Lactuca sativa* (*LsLCYe*) (accession no. LC603181) and *Tagetes erecta* (*TeLCYe*) (accession no. LC603182) were also artificially synthesized with the codons optimized to those of *E. coli* K12 strain. These LCYe fragments were inserted instead of MpLCYe to construct the plasmids pAC-HIEBI-MpLCYbΔTP-LsLCYe-Z and pAC-HIEBI-MpLCYbΔTP-TeLCYe-Z.

Artificial *CYP97C* genes from *M. polymorpha* (accession no. LC603185), *Chlamydomonas reinhardtii* (*CrCYP97C*) (accession no. LC603173), *H. pluvialis* (*HpCYP97C*) (accession no. LC603174), *Brassica napus* (*BnCYP97C*) (accession no. LC603175), *Chenopodium quinoa* (*CqCYP97C*) (accession no. LC603176), *Oryza sativa* (*OsCYP97C*) (accession no. LC603177), *L. sativa (LsCYP97C*) (accession no. LC603178), *Nicotiana tabacum* (*NtCYP97C*) (accession no. LC603179) and *Helianthus annuus* (*HaCYP97C*) (accession no. LC603180), which were designed with modified codons optimized to *E. coli* K12, were chemically synthesized. These CYP97C fragments were cloned into the pUC18 between the *Eco*RI and *Bam*HI sites.

To construct the plasmid CDF-MpLCYe, PCR was performed using the synthetic *MpLCYe* gene described above as a template. The amplified fragment was inserted into the pCDF (Merck, MO, USA), after which the *MpCYP97C* gene was also amplified and inserted into the CDF-MpLCYe, resulting in the plasmid CDF-MpCYP97C-MpLCYe.

The *crtE* gene from *Pantoea agglomerans* (accession no. ERWCRTA) was chemically synthesized without codon optimization and cloned into the pRK-HIEBI-MpLCYbΔTP-MpLCYe-Z. This plasmid was named as pRK-HIEBI-MpLCYbΔTP-MpLCYe-Z-E_Pg_.

To construct the plasmid pAC/Mev/Sciidi/Aacl/pnbA, the *Bacillus subtilis pnbA* gene (not optimized to *E. coli*) was artificially synthesized (24; accession no. BSU06089). This *pnbA* fragment was then ligated into the *Hin*dIII site in the pAC-Mev/Sciidi/Aacl (16).

The sequences of the primers used in this study are shown in Supplementary Table S3.

### 2.2 Construction of the genome modified *E. coli*

The neomycin phosphotransferase (*NPT*) gene was amplified by PCR with pKD13 (25), which contained flippase recognition target (FRT) sequences as a template. P*tac* and T*rrnB* fragments were then amplified by PCR and were inserted into the *Sal*I and *Bam*HI sites of the NPT fragments mentioned above. This NPT fragment including P*tac* and T*rrnB* was named as KDPT.

The *manX* and *manZ* genes’ fragments were then amplified by PCR with *E. coli* DNA as a template and ligated. The two distinct fragments of *yjfP* gene were also amplified and ligated. These ligated manXZ and yjfP fragments were then cloned into the pACYC184 between the *Eco*RI and *Nco*I sites. The resulting plasmids were named pAC-manXYZ and pAC-yjfP.

The KDPT fragment was digested with *Eco*RV and *Sac*I and inserted into the *Sma*I and *Sac*I sites of pAC-manXYZ and pAC-yjfP, resulting in the plasmids pAC-manXYZ-KDPT and pAC-yjfP-KDPT, respectively. The *IDI* gene fragment was amplified by PCR and inserted into the *Xho*I and *Kpn*I sites between P*tac* and T*rrnB* of the pAC-manXYZ-KDPT, yielding the plasmid pAC-manXYZ-KDPT-HI. The *Aacl-pnbA* fragment was amplified by PCR with pAC-Mev/Scidi/Aacl/pnbA as a template and inserted into the plasmid pAC-yjfP-KDPT, resulting in the plasmid pAC-yjfP-KDPT-Aacl-pnbA. The DNA fragments for the recombination were also obtained by PCR using the primers shown in Supplementary Table S2.

Either *E. coli* JM101(DE3) or JM101 (DE3) Δ(*manXYZ*)[*IDI*] carrying the Red helper plasmid pKD46 (25) was grown in SOB medium with ampicillin and 1 mM L-arabinose. The electroporation-competent cells were prepared as described previously (26). The cells were mixed with PCR fragments in an ice-cold 0.1-cm cuvette and electroporated at 1.8 kV (25 μF, 200 Ω; Gene Pulser Xcell, Bio-Rad, USA). After selection with kanamycin, the transformants were cultured overnight at 42°C and tested for ampicillin sensitivity to check for loss of the helper plasmid. Colony PCR was then performed to confirm the genome recombination.

The FLP helper plasmid pCP20 (27) was introduced into the transformants to eliminate the *NPT* gene between FRT sequences at 30°C. After selection with ampicillin resistance, the transformants were cultured at 42°C overnight and tested for kanamycin and ampicillin sensitivity to check for the loss of the *NPT* gene and helper plasmid, respectively.

### 2.3 Extraction and HPLC analysis of carotenoids from *E. coli* cells

Each transformed *E. coli* was grown in 2YT medium at 37°C until an optical density of 0.8–1.0 was achieved, induced with 0.05 mM of isopropyl β-D-thiogalactopyranoside (IPTG), and further cultured at 21°C for 2 days. Basically, we used test tubes for the bacterial cultures. When the genome-modified *E. coli* was grown, 0.1% (v/v) ethyl acetoacetate (EAA) was added together with IPTG and cultured at 21°C for 3–5 days. In the case of EAA addition, we used shake flasks for an appropriate agitation.

Extraction of carotenoids from *E. coli* was performed using the method described previously (7). *E. coli* cultures were centrifuged and cell pellets were extracted in methanol using a mixer for 5 min. Tris-HCl (50 mM, pH 7.5) (containing 1 M NaCl) was added and mixed. Then, chloroform was added to the mixture and incubated for 5 min. After centrifugation, the chloroform phase was removed and dried by centrifugal evaporation. Dried residues were resuspended with ethyl acetate and applied to the High Performance Liquid Chromatography - Photodiode Array (HPLC-PDA).

Chromatography was carried out on a Waters Alliance 2695-2996 system (Waters, Milford, MA, USA) with a column, TSKgel ODS-80Ts (4.6 × 150 mm, 5 μm; Tosoh, Tokyo, Japan), according to the method described previously (28). Briefly, the extract was eluted at a flow rate of 1.0 ml/min at 25°C with solvent A (water–methanol, 5:95, v/v) for 5 min, followed by a linear gradient from solvent A to solvent B (tetrahydrofuran-methanol, 3:7, v/v) for 5 min, solvent B alone for 8 min and then back to solvent A. Carotenoids were identified by comparing both their retention times and absorption spectra monitored using PDA relative to those of the authentic standards. For quantitative analysis, we performed the experiments three times.

### 2.4 Fermentation conditions

Cells were cultured overnight in liquid Luria Broth (LB) medium at 30°C, and then, 10 ml of the cell culture was inoculated into 1 l of modified Terrific Broth (TB) medium (per liter: 12 g Bacto Tryptone; Gibco), 24 g Bacto yeast extract, 9.4 g K_2_HPO_4_, 2.2 g KH_2_PO_4_ and appropriate antibiotics (100 mg spectinomycin, 10 mg tetracycline and 30 mg chloramphenicol). Cultures were grown at 25°C in a 3-l jar fermenter (BMJ-03P, ABLE). The pH was maintained at 7.0 by automatic addition of 28% NH_4_OH and 25% H_3_PO_4_. The agitation speed was 100 rpm. At the time of inoculation, dissolved oxygen levels were allowed to fall to 10% of O_2_ saturation with a continuous air supply of 1 volume per minute. The glucose concentration was maintained at 0.4 g/l by the addition of 15% (w/v) glucose solution. 0.1 mM IPTG and 0.1% (v/v) ethyl 3-oxobutanate were then added to the culture when Optical Density at 600 nm (OD_600_) reached 10.

### 2.5 Detection and quantification of chemical compounds

Glucose in the culture medium was analyzed by the mutarotase-glucose method using a Glucose CII Test Wako (Wako, Japan). To analyze carotenoid compounds in the culture medium, cultures were collected every 12 h by an autosampler (LA-11, ABLE). Cells were corrected by centrifugation at 5000 × g for 5 min and stored at −20°C.

Cells from 0.2 ml culture medium were homogenized with 0.5 ml acetone. About 1 ml hexane/diethyl ether (1:1) was added to acetone extract and vortexed well. Also, 1 ml water was added and mixed and then left for 10 min. The upper layer was connected to a new tube and dried in vacuum.

The extracted carotenoids were carried on an ACQUITY UPLC H-class system (Waters, Milford, CT, USA). UV–VIS absorption spectra were recorded from 200 to 500 nm using a photodiode-array detector (PDA). An Acquity 1.7 μm BEH UPLC C18 column (Waters) was also used as a stationary phase and UPLC ODS MeCN:H_2_O (85:15)–MeCN:MeOH (65:35) (linear gradient 0–15 min) as a mobile phase, at a flow rate of 0.4 ml/min.

### 2.6 Statistical analysis

For statistical analysis, we performed Student’s *t* test.

## 3. Results and discussion

Our previous study succeeded in synthesizing lutein in the transgenic *E. coli*, which has three plasmids, pACHP-Lyc, pETD-MpLCYb/MpLCYe and pRSF-MpBHY/MpCYP97C (22). However, the productivity of lutein was low (∼0.1 mg/l). To increase lutein productivity, we first tried rearranging these plasmids and made the plasmids pAC-HIEBI-MpLCYb-MpLCYe-Z and pUC -MpCYP97C. The *E. coli* with these plasmids could produce lutein, as shown below. Here, using these plasmids, we investigated the factors that affected the productivity of lutein.

### 3.1 Examination of the lycopene β-cyclase

**Figure 2.**
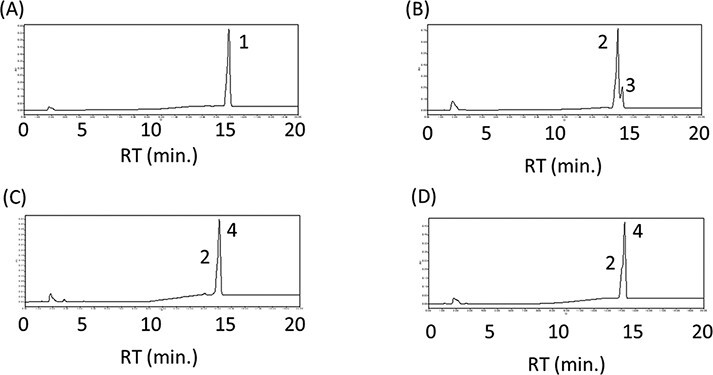
Effect of the β-monocyclase on the α-carotene production. HPLC chromatograms of the extracts from E. coli having the plasmids pAC-HIEBIYm (A), pAC-HIEBIA (B), pAC-HIEBIA-MpLCYe (C) and pAC-HIEBI-MpLCYeA (D). 1, β-carotene; 2, lycopene; 3, γ-carotene; 4, α-carotene.

Plant lycopene β-cyclases (LCYbs) are typically bicyclases, which immediately convert lycopene to β-carotene with two β-rings via γ-carotene with one β-ring. Alternatively, a few bacteria have lycopene β-monocyclase genes, for example, *cruA* from *C. phaeobacteroides* (29) and *crtYm* from a unique marine bacterium P99-3 (23). To inhibit the production of zeaxanthin (both β-rings), we investigated these lycopene β-monocyclase genes in place of the plant *LCYb*. When we introduced the plasmid pAC-HIEBIYm into *E. coli*, only the β-carotene was found (Figure 2A), which indicated that CrtYm functioned as a bicyclase like the plant LCYbs in our system. When the plasmid pAC-HIEBIA was introduced, however, the γ-carotene was detected in addition to lycopene (Figure 2B). This result showed that CruA functioned as a monocyclase as expected. Then, we constructed and transformed the plasmid either pAC-HIEBIA-MpLCYe or pAC-HIEBI-MpLCYe-A into *E. coli*. In both cases, no peak corresponding to α-carotene was found except for lycopene and δ-carotene with one ε-ring, suggesting that CruA cannot convert δ-carotene into α-carotene (Figure 2C and D). From these experiments, we found that neither CrtYm nor CruA produce α-carotene with the plant lycopene ε-cyclase (LCYe). In the future, it may be required to find the β-monocyclase, which shows high substrate specificity to δ-carotene.

**Figure 3.**
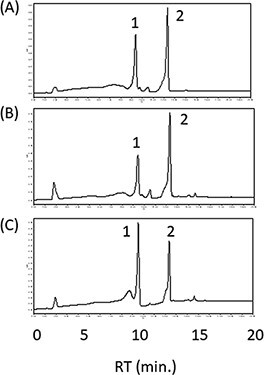
Selection of the MpLCYb. HPLC chromatograms of the extracts from *E. coli* carrying the plasmids pAC-HIEBI-MpLCYb-MpLCYe-Z (A), pAC-HIEBI-MpLCYbΔTP-MpLCYe-Z (B) and pAC-HIEBI-MpLCYbop-MpLCYe-Z (C). 1, zeaxanthin; 2, zeinoxanthin.

Next, to balance between LCYb and LCYe activities, we tried modulating the MpLCYb activity. Plant *LCYb* genes have the chloroplast transit peptides (cTPs). The existence of TPs affects their activities in *E. coli*. So, we deleted the predicted TPs from the coding region of MpLCYb (MpLCYbΔTP) and constructed the plasmid pAC-HIEBI-MpLCYbΔTP-MpLCYe-Z. In our system, it was difficult to distinguish α-carotene from β-carotene, so we compared zeinoxanthin (an α-carotene derivative) and zeaxanthin (a β-carotene derivative). In plants, the *BHY* and *CYP97A* genes function as the β-ring hydroxylase for β-carotene and zeinoxanthin, respectively. However, in *E. coli*, the bacterial CrtZ can hydroxylate both compounds with a higher activity than the plant genes *BHY* and *CYP97A*. Thus, we used *P. ananatis crtZ* for the hydroxylation of β-carotene and zeinoxanthin. In the *E. coli* having the plasmid pAC-HIEBI-MpLCYbΔTP-MpLCYe-Z, the ratio of zeinoxanthin to zeaxanthin (2.2 ± 0.1) was higher than that (1.5 ± 0.1) in the *E. coli* carrying pAC-HIEBI-MpLCYb-MpLCYe-Z (Figure 3A and B), suggesting that the deletion of TP decreased the activity of MpLCYb. Since the lycopene was not detected in the pAC-HIEBI-MpLCYbΔTP-MpLCYe-Z carrying *E. coli*, it was suggested that the activity of MpLCYbΔTP was not too weak. In contrast, when we tested the codon-optimized MpLCYb (MpLCYbop), the ratio of zeinoxanthin to zeaxanthin was 0.5 ± 0.1, indicating that the activity of MpLCYbop was higher than that of MpLCYe (Figure 3C). These results suggested that MpLCYbΔTP was most suitable to produce zeinoxanthin, the precursor of lutein.

### 3.2 Selection of the LCYe (lycopene ε-cyclase)

Our previous studies showed that the activity of the MpLCYb was stronger than that of the MpLCYe (7). Therefore, we tested several LCYes to find the stronger LCYe. We selected two *LCYe* genes from *L. sativa* (*LsLCYe*) and *T. erecta* (*TeLCYe*) in addition to *MpLCYe*. Most of the higher plants do not accumulate ε-carotene or ε-carotene derivatives such as lactucaxanthin, probably because the activities of their LCYes are not strong compared with their LCYbs. However, lettuce (*L. sativa)* accumulates lactucaxanthin with two ε-rings, and the activity of LsLCYe is considered quite strong (30). Marigold (*T. erecta*) flower is known to be rich in lutein, suggesting that the activity of TeLCYe was relatively stronger (31).

**Figure 4.**
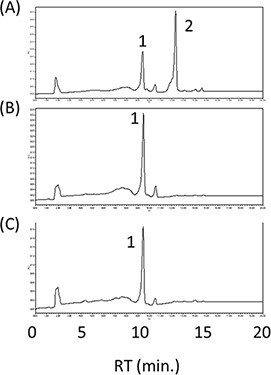
Effect of the lycopene ε-cyclase on the zeinoxanthin production. HPLC chromatograms of the extracts from *E. coli* having the plasmids pAC-HIEBI-MpLCYbΔTP-MpLCYe-Z (A), pAC-HIEBI-MpLCYbΔTP-LsLCYe-Z (B) and pAC-HIEBI-MpLCYbΔTP-TeLCYe-Z (C). 1, zeaxanthin; 2, zeinoxanthin.

For this purpose, we constructed the plasmids pAC-HIEBI-MpLCYbΔTP-LCYe-Z containing each *LCYe* gene. As a result, the peaks of zeaxanthin were predominantly detected in both cases of LsLCYe and TeLCYe (Figure 4B and C). These results indicated that both *LsLCYe* and *TeLCYe* genes did not function in *E. coli*. In contrast, the peak of zeinoxanthin was dominantly detected in the case of MpLCYe (Figure 4A). These results suggested that MpLCYe showed the highest activity among the three LCYes tested in *E. coli*. Therefore, we used the *MpLCYe* gene for further experiments.

In this study, the lettuce LCYe (LsLCYe) could synthesize ε-carotene in *E. coli*, showing its high activity (data not shown). In contrast, the MpLCYe could synthesize only δ-carotene but not ε-carotene. However, when the LsLCYe combined with MpLCYb, it did not exhibit its ability. One of the reasons is that the combination of LsLCYe and MpLCYb was not good to function together. We tried to express LsLCYb in *E. coli*, but its activity was significantly weaker than that of MpLCYb (data not shown). From these results, it is observed that the combination of MpLCYb and MpLCYe was useful in our system.

### 3.3 Screening of the *CYP97C* gene

**Figure 5.**
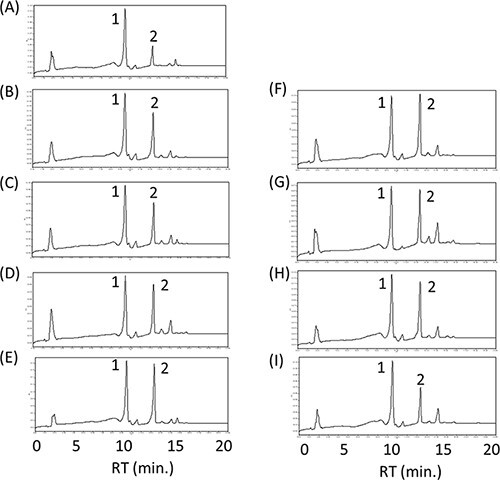
Screening of the *CYP97C* genes for the efficient lutein production. HPLC chromatograms of the extracts from *E. coli*, which have the plasmid pAC-HIEBI-MpLCYbΔTP-MpLCYe-Z with either pUC-MpCYP97C (A), pUC-CrCYP97C (B), pUC-HpCYP97C (C), pUC-BnCYP97C (D), oUC-CqCYP97C (E), pUC-OsCYP97C (F), pUC-LsCYP97C (G), pUC-NtCYP97C (H) or pUC-HaCYP97C (I). 1, lutein and zeaxanthin; 2, zeinoxanthin.

Next, we tried to find the most suitable CYP97C for the effective lutein production in *E. coli*. We selected eight *CYP97C* genes in addition to *MpCYP97C*, which we had initially used. The eight genes are from *C. reinhardtii* (*CrCYP97C*), *H. pluvialis* (*HpCYP97C*), *B. napus* (*BnCYP97C*), *C. quinoa* (*CqCYP97C*), *O. sativa* (*OsCYP97C*), *L. sativa (LsCYP97C*), *N. tabacum* (*NtCYP97C*) and *H. annuus* (*HaCYP97C*). We constructed each plasmid pUC-CYP97C and transformed it with pAC-HIEBI-MpLCYbΔTP-MpLCYe-Z into *E. coli*. As a result, in all cases, the peaks of zeinoxanthin, which did not convert to lutein, were found (Figure 5). This result suggested that the activities of these CYP97Cs were not sufficient to produce lutein exclusively. Nevertheless, the lowest peak of zeinoxanthin was observed in *E. coli* carrying pUC-MpCYP97C (Supplementary Figure S3). These results suggested that MpCYP97C was the most active CYP97C in *E. coli* among the nine CYP97Cs tested. Therefore, we used *MpCYP97C* for further experiments.

### 3.4 Addition of the *MpLCYe* gene

**Figure 6.**
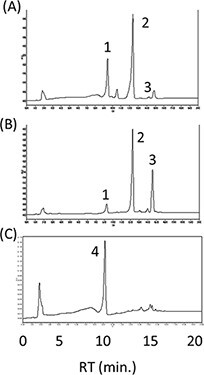
Effect of the addition of MpLCYe. HPLC chromatogram of the extracts from *E. coli* having the plasmids pRK-HIEBI-MpLCYbΔTP-MpLCYe-Z (A), pRK-HIEBI-MpLCYbΔTP-MpLCYe-Z plus CDF-MpLCYe (B) and pRK-HIEBI-MpLCYbΔTP-MpLCYe-Z plus CDF-MpCYP97C-MpLCYe (C). 1, zeaxanthin (mainly); 2, zeinoxanthin; 3, α/β-carotene; 4, lutein (mainly).

As described above, MpLCYe was suitable for the production of lutein along with MpLCYb. However, the activity of MpLCYe was weaker than that of MpLCYb. To balance the activities of β-cyclase and ε-cyclase, we added more than one copy of the *MpLCYe* gene as the plasmid CDF-MpLCYe. For the following experiments, we used the plasmid pRK-HIEBI-MpLCYbΔTP-MpLCYe-Z instead of pAC-HIEBI-MpLCYbΔTP-MpLCYe-Z. The *E. coli* with the latter plasmid showed almost the same carotenoid profile as that carrying the former one (Figure 6A). The addition of *MpLCYe* also decreased zeaxanthin and increased zeinoxanthin, suggesting the effectiveness of improving lutein production (Figure 6B). Then, to add the *MpLCYe* gene and reduce the number of plasmids, we constructed the plasmid CDF-MpCYP97C-MpLCYe. The *E. coli* carrying this plasmid and pRK-HIEBI-MpLCYbΔTP-MpLCYe-Z thus accumulated mainly lutein as expected (Figure 6C). At this point, the lutein productivity was ∼1.0 mg/l.

### 3.5 Enhancement of the upper pathway

**Figure 7.**
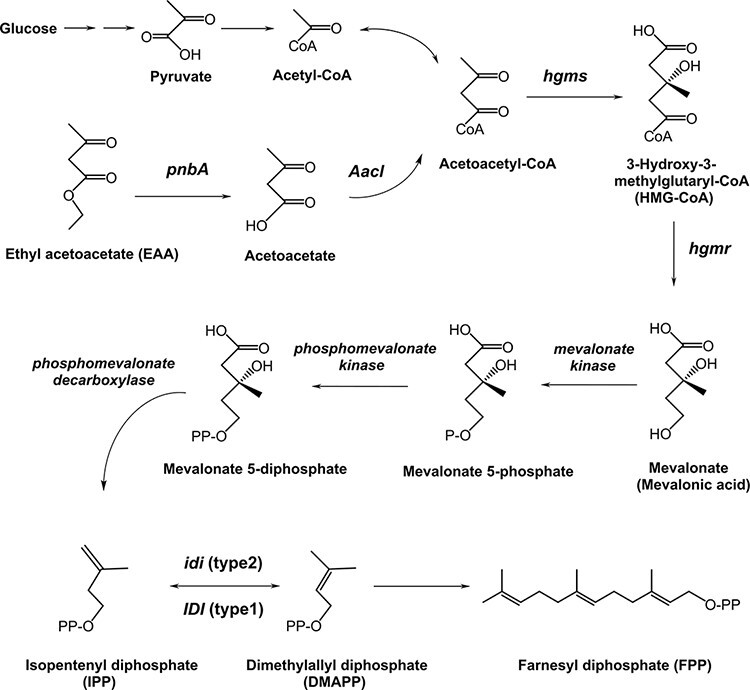
Functions of the *pnbA, Aacl* and mevalonate pathway genes introduced into *E. coli* and the relevant upstream metabolic pathway from glucose and ethyl acetoacetate (EAA) to farnesyl diphosphate (FPP). Gene names are written only for genes introduced into *E. coli. hmgs, HMG-CoA synthase; hmgr, HMG-CoA reductase.*

To increase the lutein production, we tried to enhance the upper pathway with three strategies. First, we tried integrating the *IDI* gene into the *manXYZ* region of *E. coli* chromosome (Supplementary Figure S2A). Second, we added *crtE* gene (*crtE_Pg_*) from *P. agglomerans*, which showed a higher activity than *P. ananatis*. Studies suggested that the rate limitation of carotenoid production in *E. coli* depends on the activity of *crtE* (18). Third is the insertion of Mavalonic Acid (MVA) pathway via chromosomal integration and plasmid. Many studies have proven that the addition of MVA pathway was effective for enhancing carotenoid or sesquiterpene production in *E. coli* (16, 32–40). Moreover, we can use EAA as a substrate for the MVA pathway by using the *Aacl* and *pnbA* genes to convert EAA to acetoacetyl-CoA (Figure 7) (41). The *Aacl* and *pnbA* genes were integrated into the *yjfP* region of the chromosome of *E. coli* Δ(*manXYZ*)[*IDI*] (Supplementary Figure S2B). Moreover, we introduced the plasmid pAC-Mev/Scidi/Aacl/pnbA with pRK-HIEBIMpLCYbΔTP-MpLCYe-Z-E_Pg_ and CDF-MpCYP97C-MpLCYe into *E. coli*. As a result of these strategies, the lutein productivity was improved to 2.6 mg/l.

### 3.6 Optimization of fermentation conditions for the biosynthesis of lutein

**Figure 8.**
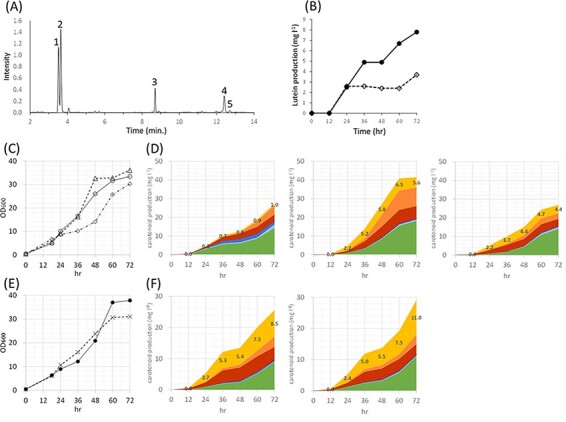
Fermentative production of lutein. (A) UPLC chromatogram of the extracts from *E. coli* strain 2 at 72 h. 1, zeaxanthin; 2, lutein; 3, zeinoxanthin; 4, α-carotene; 5, β-carotene. (B) Effect of temperature on fermentative production of lutein. 25°C, closed circle; 30°C, open square. (C) Growth curves for the previous production strain 1, open square; 2, open triangle and 3, open circle. (D) Yield of each carotenoid during fermentation of strain 1 (left), 2 (middle), 3 (right). (E) Growth curves for the production strain 2 with FeCl_3_ at the concentration of 2 mM, closed circle, and 5 mM, cross mark. (F) Effect of the adding FeCl_3_ in the culture medium of strain 2 at the concentration of 0.2 mM (left) and 0.5 mM (right). Values in the graphs in (D) and (F) showed yield of lutein (mg/l). Lutein, yellow; zeinoxanthin, orange; α-carotene, red; zeaxanthin, green; β-cryptoxanthin, light blue; β-carotene, blue; lycopene, purple.

Finally, to improve the yield of lutein, the fed-batch fermentation technique was applied. Figure 8A shows the chromatogram of carotenoids extracted from *E. coli* cells. Many carotenoids, especially lutein and zeaxanthin, were separated by Ultra Performance Liquid Chromatography (UPLC). The results of aerobic batch and continuous cultivations of *E. coli* strains indicated that less acetate was accumulated (data not shown) with a higher lutein yield at 25°C as compared to the case at 30°C (Figure 8B). As a result of comparing the IPTG concentrations between 0.1 mM and 0.2 mM, the ratio of zeaxanthin was extremely high in 0.2 mM IPTG (data not shown), which was not preferable for lutein synthesis. Therefore, 0.1 mM IPTG was used as an induction condition for gene expression.

The productivity of lutein by jar fermenter was compared between three strains of strain 1 (pRK-HIEBI-MpLCYb-MpLCYe-Z + pAC-Mev/Scidi/Aacl/pnbA + CDF-MpCYP97C-MpLCYe + pETD-MpLCYb/JM101(DE3) Δ(*manXYZ*)[*IDI*] Δ(*yjfP*)[*Aacl-pnbA*]), strain 2 (pRK-HIEBI-MpLCYbΔTP-MpLCYe-Z-E_Pg_ + pAC-Mev/Scidi/Aacl/pnbA + CDF-MpCYP97C-MpLCYe/JM101(DE3) Δ(*manXYZ*)[*IDI*] Δ(*yjfP*)[*Aacl-pnbA*]) and strain 3 (pRK-HIEBI-MpLCYb-MpLCYe-Z-E_Pg_ + pAC-Mev/Scidi/Aacl/pnbA + CDF-MpCYP97C-MpLCYe/JM101(DE3) Δ(*manXYZ*)[*IDI*] Δ(*yjfP*)[*Aacl-pnbA*]) (Figure 8C and D). Strain 2 showed the highest carotenoid productivity and the highest lutein yield of 6.5 mg/l. Since it is known that CYP97C, a key enzyme of lutein synthesis, contains heme (42), we investigated whether the addition of FeCl_3_ to the fermentation medium contributed to the increase in lutein yield. Results showed that the addition of FeCl_3_ maximized the yield of lutein, and in particular, when 0.5 mM FeCl_3_ was added, the productivity of lutein was 11.0 mg/l (Figure 8E and F).

### 4. Conclusion

So far, we have produced lutein in *E. coli* by metabolic engineering (22); however, its productivity was low (∼0.1 mg/l; our unpublished data). Indeed, no reports have been published describing the yield of lutein biosynthesized in the metabolically engineered *E. coli*. In this study, we applied several strategies such as the rearrangement of the plasmid, selecting the proper genes and enhancing the upper pathway. As a result, we achieved a yield of 11 mg/l. However, the intermediates (zeinoxanthin and α-carotene) or by-products (zeaxanthin) remained. For the higher lutein productivity, it will be required to employ enzymes with higher catalytic activities and to enhance the introduced genes’ expressions. In our experiments, the lutein produced by *E. coli* is almost present as free form, whereas most lutein in the marigold flowers or other plants are esterified, which suggests that lutein obtained from the genetically engineered *E. coli* could be advantageous for purification and commercialization.

## Supplementary Material

ysab012_Supp

## Data Availability

Data and materials used in this study are available on request to the corresponding author.
