# Supplementary material for: Pathway engineering for high-yield production of lutein in Escherichia coli
Source: Synth Biol (Oxf). 2021 May 15;6(1):ysab012. doi: 10.1093/synbio/ysab012 (PMC8546607; doi:10.1093/synbio/ysab012)
Supplement: ysab012_Supp [file ysab012_supp.zip › LuteinSuppRev1.docx]

Supplementary Table 1.　The plasmids constructed in this study

| Plasmid name | Description |
| --- | --- |
| pAC-HIEBI | H. pluvialis IDI, P. ananatis crtE, crtB and crtI |
| pRK-HIEBI | H. pluvialis IDI, P. ananatis crtE, crtB and crtI |
| pAC-HIEBI-MpLCYb*1 | IDI, crtE, crtB, crtI and MpLCYb*1 |
| pRK-HIEBI-MpLCYb*1 | IDI, crtE, crtB, crtI and MpLCYb*1 |
| pAC-HIEBIYm | IDI, crtE, crtB, crtI and P99-3 crtYm |
| pAC-HIEBIA | IDI, crtE, crtB, crtI and C. phaeobacteroides CruA |
| pAC-HIEBIA-MpLCYe | IDI, crtE, crtB, crtI, MpLCYe and CruA |
| pAC-HIEBI-MpLCYe-A | IDI, crtE, crtB, crtI, MpLCYe and CruA |
| pAC-HIEBI-MpLCYb*1-MpLCYe-Z | IDI, crtE, crtB, crtI, crtZ, MpLCYb*1 and MpLCYe |
| pRK-HIEBI-MpLCYbΔTP-MpLCYe-Z | IDI, crtE, crtB, crtI, crtZ, MpLCYbΔTP and MpLCYe |
| pAC-HIEBI-MpLCYbΔTP-LCYe*2-Z | IDI, crtE, crtB, crtI, crtZ, MpLCYbΔTP and LCYe*2 |
| pUC-CYP97C*3 | CYP97C*3 |
| CDF-MpLCYe | MpLCYe |
| CDF-MpCYP97C-MpLCYe | MpCYP97C, MpLCYe |
| pAC-Mev/Scidi/Aacl/pnbA | Mevalonate pathway gene cluster, Scidi, Aacl, pnbA |

*1, LCYb indicates either MpLCYb, MpLCYbΔTP, or MpLCYbop

*2, LCYe indicates either LsLCYe or TeLCYe

*3, CYP97C indicates either MpCYP97C, CrCYP97C, HpCYP97C, BnCYP97C, CqCYP97C, OsCYP97C, LsCYP97C, NtCYP97C or HaCYP97C

Supplementary Table 2. Strains used in this study

| Strain | Plasmids |
| --- | --- |
| JM101(DE3) | pAC-HIEBIYm (Fig. 2) |
|  | pAC-HIEBIA (Fig. 2) |
|  | pAC-HIEBIA-MpLCYe (Fig. 2) |
|  | pAC-HIEBI-MpLCYe-A (Fig. 2) |
|  | pAC-HIEBI-MpLCYb*1-MpLCYe-Z (Fig. 3) |
|  | pRK-HIEBI-MpLCYbΔTP-MpLCYe-Z (Fig. 6) |
|  | pAC-HIEBI-MpLCYbΔTP-LCYe*2-Z (Fig. 4) |
|  | pAC-HIEBI-MpLCYbΔTP-MpLCYe-Z + pUC-CYP97C*3 (Fig. 5) |
|  | pRK-HIEBI-MpLCYbΔTP-MpLCYe-Z + CDF-MpLCYe (Fig. 6) |
|  | pRK-HIEBI-MpLCYbΔTP-MpLCYe-Z + CDF-MpCYP97C-MpLCYe (Fig. 6) |
|  |  |
| JM101(DE3) | pRK-HIEBI-MpLCYb-MpLCYe-Z + pAC-Mev/Scidi/Aacl/pnbA + CDF-MpCYP97C-MpLCYe + pETD-MpLCYb |
| Δ(manXYZ)[IDI] | pRK-HIEBI-MpLCYbΔTP-MpLCYe-Z-E_Pg_ + pAC-Mev/Scidi/Aacl/pnbA + CDF-MpCYP97C-MpLCYe |
| Δ(yjfP)[Aacl-pnbA] | pRK-HIEBI-MpLCYb-MpLCYe-Z-E_Pg_ + pAC-Mev/Scidi/Aacl/pnbA + CDF-MpCYP97C-MpLCYe |

*1, LCYb indicates either MpLCYb, MpLCYbΔTP, or MpLCYbop

*2, LCYe indicates either LsLCYe or TeLCYe

*3, CYP97C indicates either MpCYP97C, CrCYP97C, HpCYP97C, BnCYP97C, CqCYP97C, OsCYP97C, LsCYP97C, NtCYP97C or HaCYP97C

Supplementary Table 3. Primers used in this study

| Prime name | Sequence |
| --- | --- |
|  |  |
| Construction of the expression plasmids | |
| PtacF1 | GAAGCTTAGCTGTTGACAATTAATC |
| PtacR1 | AGGTACCGCTCGAGTGTTTCCTGTGTGAAATTG |
| TrrnBF1 | CGGTACCCTGTTTTGGCGGATGAGAG |
| TrrnBR1 | CAGATCTCTGCTTTCCTGATGCAAAAAC |
| CrtYmF | AGGATCCAGGAGGCAGCTATGGATAATCATTACGATATA |
| CrtYmR | CGGTACCCAGATCTTAAAGAGACTTAAAAAAG |
| MpLCYbF1 | CGGATCCAGGAGGCAGCTATGAGCTCGACGAGGTATGG |
| MpLCYbF2 | CGGATCCAGGAGGCAGCTATGAAGAGTAAGGCTGTGGATC |
| MpLCYbR | AGGTACCCAGATCTTATTGCCGCTTCATCAATG |
| CrtZF | CGGATCCAGGAGGCAGCTATGTTGTGGATTTGGAATG |
| CrtZR | AGGTACCCAGATCTTACTTCCCGGATGCGGG |
| MpCYP97CF1 | GGAATTCGATGAGTGATATGGAGAAAGAATC |
| MpCYP97CF2 | GCCATGGGTGATATGGAGAAAGAATC |
| MpCYP97CR | CGGTACCCAGATCTTAAATACTCGCTAATTCGGCG |
| MpLCYeF | GCATATGGGAACTATTGACCGTGC |
| MpLCYeR | CGGTACCCAGATCTTAACGCA |
|  |  |
| Construction of the plasmids for genome recombination | |
| manXF | GCAATTGGAGGTAGCAAGTGACC |
| manXR | CGAGCTCATCCCGGGACGACAATGCGGCTGGC |
| manZF | CGAGCTCCACTGGCTGGTGTAGGC |
| manZR | CCCATGGCCTGGCATTAACTGGTC |
| yjfPF1 | CCAATTGAATTGTTACCTGGTACGC |
| yjfR1 | CGAGCTCATCCCGGGTTGTAGCAAGATTTGCCAG |
| yjfPF2 | CGAGCTCGTCGATGGGCGCGATGAC |
| yjfPR2 | CCCATGGAGATGCTGGCGGAAAAATG |
| pKD13F | GGATATCATTACACGTCTTGAGCG |
| pKD13R | GGAGCTCGATCAGTGATAAGCTGTC |
| PtacF2 | CGTCGACCTGTTGACAATTAATC |
| TrrnBR2 | GGGATCCTGCTTTCCTGATGCAAAAAC |
| AaclF | ACTCGAGGAGGCAGCTATGTCCAAGCTGGCACGGCTTG |
| pnbAR | CGGTACCTTATTCGCCTTTGCTCGGAAAC |
|  |  |
| Amplification of the DNA fragment for genome recombination | |
| manXF |  |
| manZR |  |
| yjfPF1 |  |
| yjfPR2 |  |
| manXgR | CAGCGGGTGGCGACCTGACC |
| manZgF | CGAACGTATGCAGGCACTGG |
| yjfPgR | CTTCGGCTATTGCCGCACG |


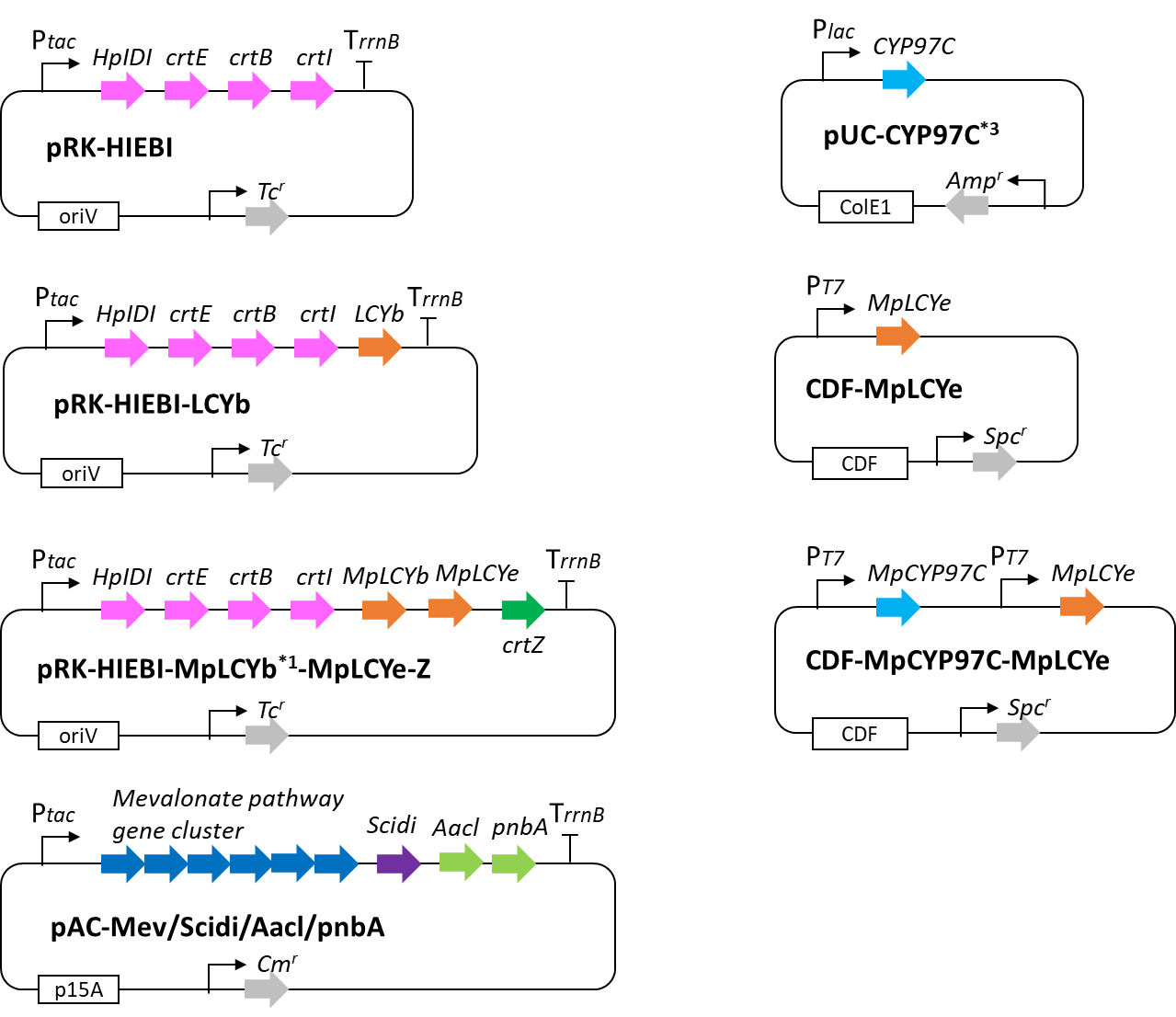

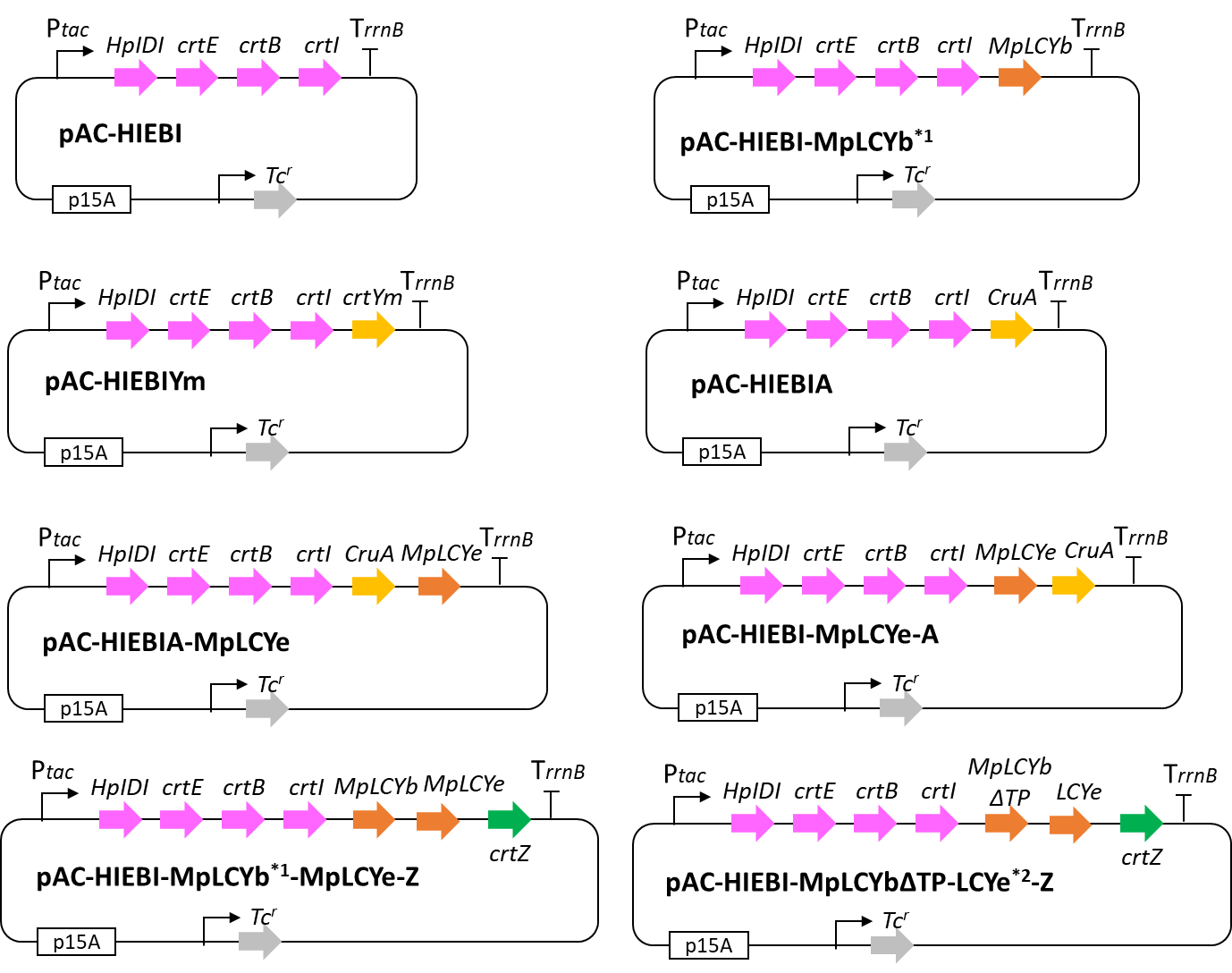
Supplementary Figure 1. The plasmids constructed in this study.


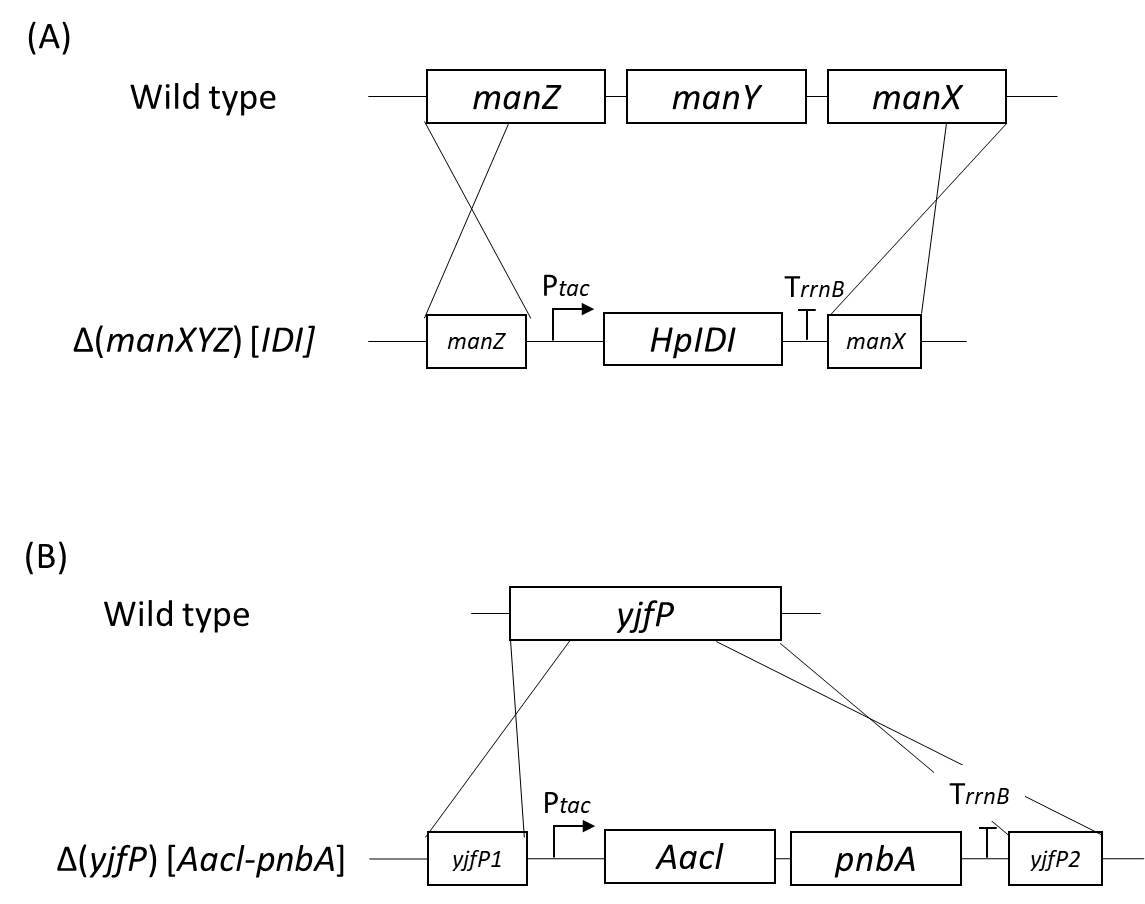
Supplementary Figure 2. The genome recombination.


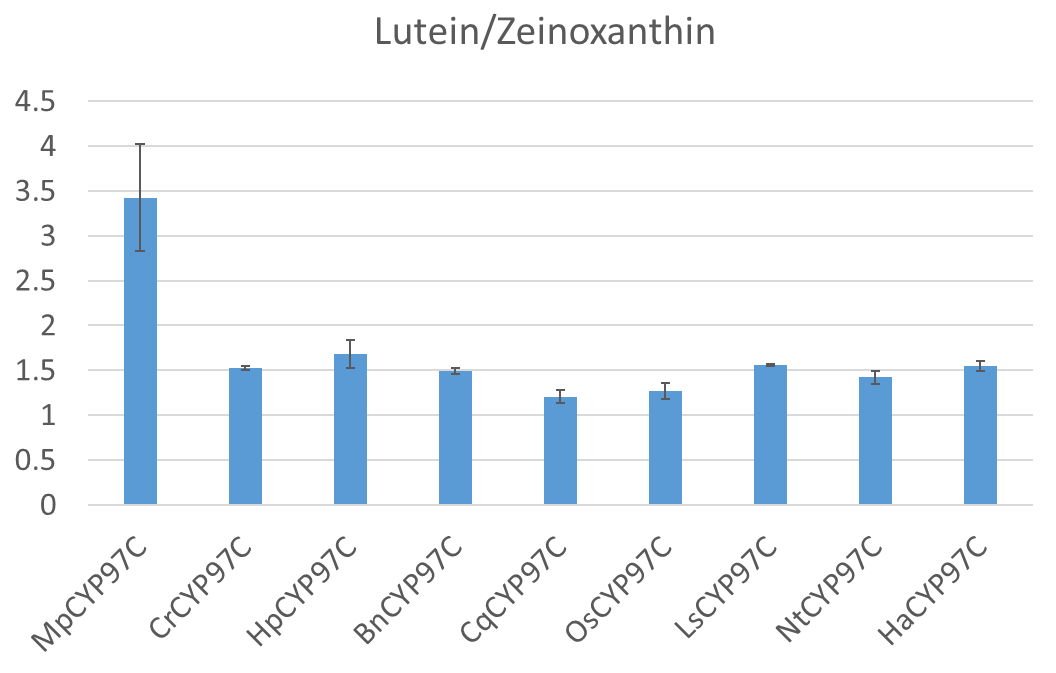


Supplementary Fig. 3. The comparison of several CYP97Cs

The rations of lutein to zeinoxanthin are shown. Data are average ± SD of three independent measurements. ***; P<0.01.
